# THE SHORT-TERM EFFICACY OF HEAT THERAPY FOR OSTEOARTHRITIS OF THE HANDS: A SYSTEMATIC REVIEW AND META-ANALYSIS

**DOI:** 10.2340/jrm.v58.45883

**Published:** 2026-08-10

**Authors:** Kai CHENG, Zhongyi ZHANG, Jiaju ZHOU, Yi TANG, Wen ZHANG, Xinyu HU, Fangjie GUO, Peijian TONG, Shuaijie LV

**Affiliations:** Center of Orthopedics and Traumatology, The First Affiliated Hospital of Zhejiang Chinese Medical University (Zhejiang Provincial Hospital of Chinese Medicine), Hangzhou, Zhejiang, China

**Keywords:** osteoarthritis of the hand, heat therapy, meta-analysis, randomized controlled trials, pain management

## Abstract

**Objective:**

To systematically evaluate the clinical efficacy of heat therapy for hand osteoarthritis.

**Design:**

Systematic review and meta-analysis of randomized controlled trials (RCTs).

**Subjects/Patients:**

9 RCTs (*n* = 627) from 13 identified studies included in meta-analysis.

**Methods:**

Databases were searched up to 19 December 2025. Risk of bias was assessed via RoB 2.0. Standardized mean differences (SMD) and meta-regression were calculated using Stata 17.0.

**Results:**

Heat therapy significantly reduced Visual Analog Scale (SMD = −0.60, *p* = 0.01), Australian/Canadian Osteoarthritis Hand Index – Pain Score and Health Assessment Questionnaire (*p* < 0.05), and improved grip strength (SMD = 0.43, *p* = 0.01). No significant changes were observed for other outcome indicators. Subgroup analysis showed multimodal therapy (paraffin: heat + mechanical compression; mud: heat + chemical/mechanical stimulation) outperformed monotherapy (heated gloves: pure thermal) for pain (SMD = –0.85) and grip strength (SMD = 0.55). Additionally, meta-regression analysis found that longer single treatment duration (*p* = 0.03) and higher weekly treatment frequency (*p* = 0.04) were key moderators for better grip strength recovery.

**Conclusion:**

Heat therapy, especially multimodal approaches, effectively alleviates short-term pain and improves grip strength in hand osteoarthritis. Clinicians may ensure adequate treatment frequency and duration to optimize outcomes.

## LAY ABSTRACT

Many people living with hand osteoarthritis struggle with daily tasks like opening jars or buttoning shirts due to persistent pain and weak grip strength. Our study looked at whether heat therapy – using tools like paraffin wax, mud, or heated gloves – actually helps. After reviewing the latest research to the end of 2025, we found that heat therapy is a safe and effective way to reduce hand pain and make finger movement easier. Importantly, we discovered that “multimodal” treatments (like wax) work better than simple heated gloves. We also found that the benefit depends on “dosage”: for the best results in regaining hand strength, patients need to use heat therapy often enough (> 5 times/week) and for a long enough time (> 20 min per session). For clinical practice, this means heat is more than a comfort measure – it is a functional tool that can help patients stay active and independent without relying solely on medication.

Hand osteoarthritis (HOA) is a common form of osteoarthritis, typically characterized by symptoms such as intermittent pain, morning stiffness, and limited joint mobility (1). X-rays reveal narrowing of the joint space, the formation of osteophytes, subchondral sclerosis, and the formation of subchondral cysts (2). Epidemiological data show that the hands are the second most common site of osteoarthritis, after the knees. It is estimated to affect around 142 million people worldwide (3). This condition is most commonly seen in women aged 55 and over (2, 4). Furthermore, radiological diagnosis of osteoarthritis is more common than clinical diagnosis. Risk factors for osteoarthritis of the hands include advancing age, being female, a family history of osteoarthritis, repetitive use of the hands, obesity, metabolic syndrome, and systemic low-grade inflammation (5, 6). As the global population ages at an accelerating rate, the burden of hand osteoarthritis is expected to increase further, making it a public health issue that cannot be ignored.

Although osteoarthritis of the hands is a common condition, treatment options for patients have remained limited for many years (7). Heat therapy has long been applied in the rehabilitation of musculoskeletal disorders. In recent years, high-quality evidence has been accumulating, demonstrating its potential value in relieving pain and improving function (8, 9). Heat therapy often includes treatments such as mud baths, paraffin wax baths, and heated gloves, all of which induce a thermal adaptation effect in local tissues. These treatments raise local temperatures, relax muscles, and dilate blood vessels, thereby alleviating pain, reducing chronic inflammation, increasing the elasticity of connective tissue, and accelerating metabolism (10, 11).

At the clinical level, thermotherapy has gained some recognition. The 2019 American College of Rheumatology (ACR) guideline explicitly recommends paraffin bath therapy as a non-pharmacological treatment option for hand osteoarthritis (12), whereas the French Society of Rheumatology includes a broader range of heat therapies (including paraffin baths, mud baths, etc.) in its recommendations (13). However, although thermotherapy is widely used in clinical practice and recommended in guidelines, the current evidence base remains significantly limited. A systematic review published by Ye et al. in 2011 evaluated the efficacy of rehabilitation interventions, including thermotherapy, in hand osteoarthritis; however, the review included only a limited number of studies on thermotherapy, and its conclusions were based on a single small-sample trial, resulting in insufficient evidence strength (14). Meanwhile, although a 2025 systematic review (15) has reconfirmed the potential value of heat therapy in the management of hand osteoarthritis, the development of wearable heating technologies necessitates more in-depth quantitative analysis of these single physical factors to optimize clinical dosage standards. At the same time, research into the use of heat therapy for rheumatic and musculoskeletal conditions is expanding from the evaluation of clinical efficacy to the elucidation of molecular mechanisms; this multidimensional research perspective provides new evidence for elucidating the clinical benefits of physical therapy (9).

In light of recent advances in the field of thermotherapy, this study synthesizes the latest evidence from randomized controlled trials (RCTs), including that on wearable heating technologies, and provides an in-depth analysis of the differences in clinical benefits associated with various heating modalities. Against this backdrop, this systematic review and meta-analysis aims to systematically evaluate the therapeutic value of heat therapy in addressing pain and functional limitations in patients with osteoarthritis (OA) through rigorous quality assessment and pooling of effect sizes, with the aim of providing clinicians, physical therapists, and related rehabilitation professionals with evidence that is both forward-looking and practical.

## METHOD

This systematic review adheres to the recommendations of the PRISMA statement on preferred reporting items for systematic reviews and meta-analyses and complies with the Guidelines for Quality of Methodology in Systematic Reviews. This systematic review and meta-analysis have been registered with PROSPERO (CRD420261320313).

### Information sources and search strategy

To implement a comprehensive search strategy, we systematically searched electronic databases for relevant published studies, including PubMed, Web of Science, the Cochrane Library and Embase. The search was conducted by combining subject headings and free-text terms. Search terms were adapted to suit each specific database. The final search date was from the inception of each database to 19 December 2025. The detailed search strategy is provided in Table SI. The reference lists of all selected articles were independently screened to identify any additional studies that may have been omitted from the initial search.

### Eligibility criteria

In accordance with the Population, Intervention, Comparison, Outcome and Study Design (PICOS) framework used to define the inclusion criteria for this study, the inclusion criteria for the studies included in our meta-analysis were carefully designed as follows.

Inclusion criteria:

1. Population: Patients definitively diagnosed with hand osteoarthritis with no restrictions regarding gender, age, or duration of the condition. Furthermore, the eligibility criteria for participants must be reported in the original study.
2. Intervention: Acceptable types of intervention in the intervention group may include any form of heat therapy, such as paraffin wax baths, mud baths, or heated gloves.
3. Comparison: Eligible control types in the control group may include standard care, sham treatment/placebo interventions (for example, sham mud baths or sham paraffin baths – using similar media at room temperature to simulate the treatment process without providing thermal stimulation), and active-controlled studies (active-controlled studies are included in the systematic review only to support overall efficacy and are not included in the meta-analysis).
4. Outcomes: The outcomes analysed in the studies include measures that assess hand pain and function, such as VAS, grip strength, and AUSCAN.
5. Study design: Only randomized controlled trials (RCTs) to be included.

Exclusion criteria: (*i*) any non-heat therapies; (*ii*) animal or cell studies; (*iii*) non-randomized controlled trials (RCTs); (*iv*) studies with missing or unavailable data.

### Selection and data collection process

All report titles and abstracts were carefully screened, and the eligibility of full-text studies was comprehensively assessed against established inclusion and exclusion criteria. Reports deemed eligible underwent a thorough review, and relevant data were systematically recorded. Prior to commencing data extraction, both researchers underwent standardized training to enable them to independently extract data from the 5 studies and calibrate their consistency. Subsequently, both researchers independently screened the literature, extracted data, and cross-checked these against the inclusion and exclusion criteria. In the event of disagreement, discussions were held to resolve the issue, and a third investigator was consulted where necessary. The kappa coefficient was reported to indicate inter-rater agreement. Where study information was incomplete, authors were contacted to obtain further details. Information was extracted using a pre-designed data extraction form. Key information extracted from the data included: basic study details (first author, year of publication), participant information (sample size, gender distribution, mean age), intervention details (intervention group measures, control group measures, heating details), methodological information (study design, time points for short-term outcome measurements) and outcomes.

We defined the short-term follow-up period for hand osteoarthritis as 3 months or less. If a trial reported data at multiple time points, we selected the time point closest to 3 months. For each outcome, the mean and standard deviation (SD) were collected for each measurement point. If a study did not report relevant data for extraction, we contacted the corresponding author to obtain any missing numerical outcome data. For studies that did not report the standard deviation of the endpoint, we followed the Cochrane Handbook for Systematic Reviews of Interventions (Version 6.3, 2022) to estimate this value. Based on the reported baseline standard deviation and the standard deviation of the change, we assumed a correlation coefficient of 0.5 between the baseline value and the change, using the formula shown in Appendix S1, and tested the impact of this assumption via sensitivity analysis (16). For studies reporting only the median and range or interquartile range, we estimated the mean and standard deviation using the optimization formula proposed by Wan et al. (17), depending on the specific circumstances of the data reporting. All results are rounded to 2 decimal places; see Appendix S2 for details. For outcome measures requiring pooled data (such as left-hand grip strength and right-hand grip strength), we used the formula recommended in the Cochrane Handbook (Appendix S3) to reconstruct the overall mean and standard deviation for the study from the means, standard deviations and sample sizes of each subgroup; the reconstructed data were then included as a single study in the meta-analysis.

### Outcome indicator

The outcome measures were pain and function. The primary outcome measures in this study were the VAS and the total AUSCAN score. Secondary outcomes included scores for individual AUSCAN subscales (pain, stiffness, function), grip strength, pinch strength, and HAQ scores. All meta-analyses were conducted using Stata version 17.0 (StataCorp LLC, College Station, TX, USA), in accordance with Cochrane guidelines. As the included trials used different scales to assess the same outcomes, standardized mean differences (SMD) were calculated for continuous data using 95% confidence intervals.

### Study risk of bias assessment

The 2 review authors independently assessed the risk of bias in the trials using the Cochrane Risk of Bias Tool (RoB 2, Version 2) from the Cochrane Handbook. We assessed the risk of bias in the following domains: the randomization process, deviation from the intended intervention, missing outcome data, measurement of outcomes, and selection of results for reporting. These domains were classified as having a high, low, or unclear risk of bias. The overall risk of bias was based on the assessments across all domains and typically corresponded to the most serious risk of bias identified in any single domain. Any disagreements were resolved through discussion or arbitration.

### Statistical analysis

The analysis was conducted using the statistical software Stata 17.0. In accordance with the Cochrane Handbook, we employed a random-effects model for outcome measures with heterogeneity exceeding 50%, and a fixed-effects model for those with heterogeneity below 50%; this enabled us to obtain pooled estimates for all outcome measures. *P* < 0.05 was considered statistically significant for the overall effect. Furthermore, to further explore potential sources of heterogeneity, we conducted pre-specified subgroup analyses, categorizing studies into multimodal thermotherapy and monotherapy groups based on the method of heat application. Among them, the multimodal heat therapy group refers to heat therapy combined with at least 1 additional physical stimulus (mechanical, chemical), such as paraffin therapy and mud baths. The monotherapy group relied primarily on the thermal effect alone, including hot compresses and heated gloves. Control groups were categorized according to intervention type into placebo control groups and standard care control groups. Concurrently, we performed univariate meta-regression analyses using 5 pre-specified covariates: age, sample size, heat therapy temperature, duration of a single treatment session, and frequency of treatment per week. *P* < 0.05 indicated that the variable was statistically significant. The associated variables were highlighted and interpreted in the discussion section. The 2 methods complemented each other, aiming to explain heterogeneity from different perspectives.

We used sensitivity analysis to assess the robustness of the results. During the selection of statistical methods, we applied random-effects or fixed-effects models to assess the overall effect. Furthermore, we assessed the robustness of the results by sequentially excluding studies and then re-conducting the meta-analysis. A significant change in the pooled effect size following the exclusion of a specific study indicated a significant impact on the overall results.

### Reporting bias assessment

Although the Cochrane Handbook recommends conducting an analysis of publication bias based on the results of 10 or more studies, we employed the Egger test because each included outcome involved ≥ 5 studies and the total number of participants exceeded 250; as this is below the threshold, the test has been shown to maintain acceptable statistical power and a Type I error rate. The test result was *P* > 0.05, indicating no significant small-study effect.

### Certainty assessment

To assess the certainty of the evidence, we employed the Grading of Recommendations, Assessment, Development and Evaluation (GRADE) methodology. Two independent assessors used the GRADEpro GDT software (gradepro.org) to classify the certainty of the evidence as very low, low, moderate, or high. We have provided footnotes explaining the reasons for downgrading the evidence, including risk of bias, inconsistency, indirectness, imprecision, and publication bias.

### Supplementary analysis

With regard to the formula in Appendix S1 we set *r* to 0.3 and 0.7 respectively to investigate whether the conversion of the formula introduces any bias in the results (18).

## RESULTS

### Literature search results

**Fig. 1:**
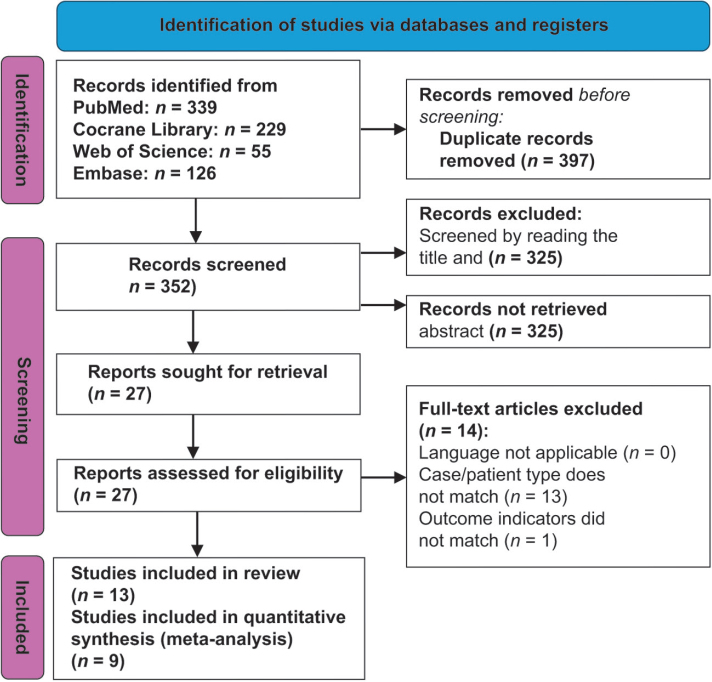
Research selection process.

This study initially identified a total of 749 articles: 339 from PubMed, 126 from Embase, 55 from Web of Science, and 229 from the Cochrane Library. After excluding duplicate entries, 352 studies remained. The titles and abstracts were then reviewed, and 27 articles were selected for further analysis. Ultimately, this systematic review included 13 studies, with 9 of these included in the meta-analysis; the kappa score for the review process was 0.86, indicating a high degree of agreement among the researchers. The screening procedure and corresponding results are detailed in the PRISMA flowchart (Fig. 1).

### Study characteristics and quality assessment

Table I describes the characteristics and details of the studies included in the review (19–31). Nine of these studies were subject to a meta-analysis (20–27,29), A total of 627 patients were involved. All studies were RCTs. Figs. S1 and S2 summarizes the risk of bias in each study and the overall risk of bias. Four articles were classified as “high risk”, 3 as “some concerns”, and 6 as “low risk”.

**Table I:** Characteristics and details included in the study

Author	Year	Sample size (intervention/control)	Female (Intervention/control)	Age (Intervention/control)	Intervention	Control	Design	Short-term outcome measurement point	Long-term outcome measurement point	Outcome	Treatment temperature (℃)	Duration of a single treatment (min)	Treatment sessions*n/*week	Total treatment duration (min)
Aksanyar	2022	40/34	no report	57.72 ± 7.03/55.15 ± 8.31	Peloid group	Paraffin group	RCT	1 month	No report	VAS, AUSCAN, HAQ, grip strength, SF-36				
Aksoy#	2017	30/25	29/22	58.17 ± 9.26/60.6 ± 8.52	Peloid therapy ± home exercise programme	Home exercise programme	RCT	1 month	No report	VAS, AUSCAN, HAQ, grip strength, pinch strength	47	30	5	300
Bartholdy#	2024	100/100	86/87	72 ± 8.8/71 ± 9.7	Heated mittens	Mittens without heat	RCT	6 weeks	No report	VAS, AUSCAN, grip strength		37	7	1554
De Azevedo#	2023	34/33	32/31	59 ± 10/60 ± 9	Paraffin baths	Leaflet with information for protection of hand joints	RCT	12 weeks	No report	VAS, grip strength, pinch strength, CHFS, FIHOA, SF-12		20	3	240
Dilek#	2013	24/22	20/20	58.87 ± 9.47/59.95 ± 8.71	Paraffin bath therapy ± theoretical information	Theoretical information	RCT	3 months	No report	VAS, AUSCAN, DFI, ROM, grip strength, pinch strength, painful and tender joint counts, paracetamol intake	50	15	3	225
Fioravanti#	2014	30/30	28/24	72.43 ± 8.26/69.23 ± 9.91	Mud packs and generalized thermal baths	Regular outpatient care routine	RCT	3 months	12 months	VAS, FIHOA, HAQ, SF-36	Combination of mud packs applied on both hands for 20 min at an initial temperature of 43 °C and with a sulphate-calcium-magnesium-fluorides water at 38°C for 15 min in a bathtub	35	6	35*12
Gyarmati#	2017	23/24	22/23	64.9 ± 4.4/64.0 ± 4.7	hévíz mud	Fake hévíz mud	RCT	3 weeks	16 weeks	VAS, hand grip strength, swollen and tender joint count, duration of morning joint stiffness, HAQ, EQ-5D-3L	42	20	5	300
Horváth#	2012	21/21	17/18	63.5 ± 4.7/63.8 ± 4.4	Bathed in thermal mineral water ± magnetotherapy	Magnetotherapy	RCT	13 weeks	No report	VAS, grip strength, pinch strength, the number of swollen and tender joints of the hand, the duration of morning joint stiffness, HAQ, SF-36	38	20	5	300
Kasapoğlu Aksoy#	2018	31/30	28/25	57.2 ± 10.6/61.3 ± 8.4	Paraffin therapy ± home-based exercise programme	Home-based exercise programme	RCT	6 weeks	Nno report	VAS, AUSCAN, HAQ, grip strength, pinch strength	52	20	5	200
Öncel	2021	36/41	29/40	61.9 ± 10.3/64.2±10.3	Paraffin bath therapy	Fluidotherapy	RCT	3 months	No report	VAS, DHI, Grip strength, SF-36				
Savaş#	2019	29/20	28/17	64.82 ± 8.32/65.50 ± 9.21	Hot compress ± routine pharmacological treatment	Routine pharmacological treatment	RCT	15 days	No report	VAS, AUSCAN	40~45	20–30	7	140~210
Uçar	2011	29/29	29/29	58.83 ± 7.21/56.83 ± 8.28	Whirlpool treatment	Paraffin treatment	RCT	12 weeks	No report	VAS, AUSCAN, SF-12, grip strength				
Ustun	2023	21/21	No report	60.43 ± 7.36/59.52 ± 6.92	Paraffin wax	Prolotherapy	RCT	3 months	No report	VAS, DHI, Grip strength, Pinch strengths				

# Studies included in the meta-analysis.

### Studies included in the meta-analysis

**Fig. 2:**
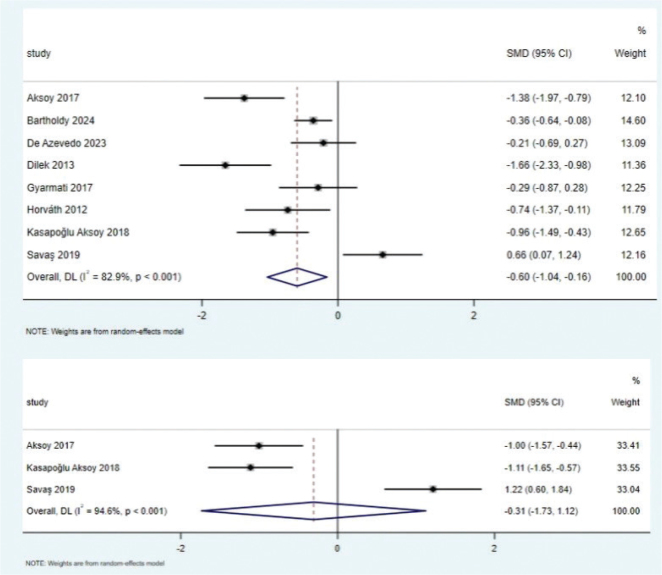
Forest plot of VAS and AUSCAN.

**Fig. 3:**
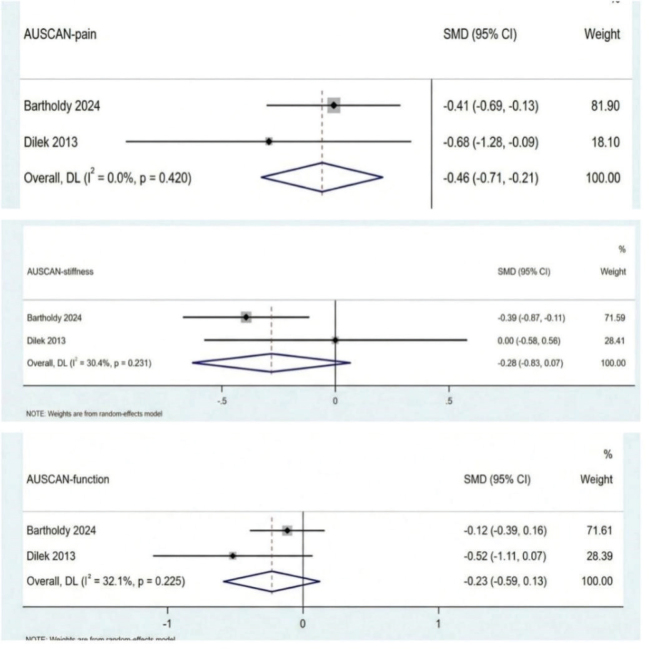
Forest plot of AUSCAN sub-items.

**Fig. 4:**
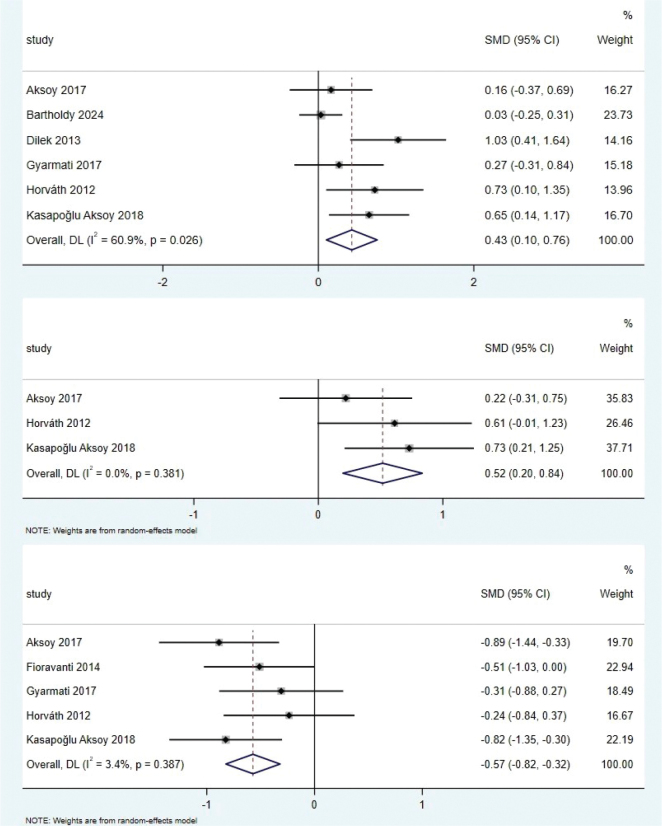
Forest plot of grip strength–pinch strength and HAQ.

*Meta-analysis results.* Table II provides a detailed overview of the results of the meta-analysis. The main findings show a high degree of heterogeneity in both the VAS and AUSCAN. The VAS scores indicated that the scores in the heat therapy group were significantly lower than those in the control group (*p* = 0.01). There was no significant difference in AUSCAN scores between the heat therapy group and the control group (*p* = 0.67). Within the AUSCAN subscales, only the AUSCAN-pain score showed a significant difference between groups (*p* = 0.00). Heat therapy resulted in more significant improvements in both grip strength and pinch strength scores (*p* < 0.05). The heat therapy group also achieved significantly higher HAQ scores than the control group (*p* < 0.05). See Figs. 2–4 for the detailed forest plots.

**Table II:** Detailed results of the meta-analysis

Outcome	Total SMD (95% CI)	*p*-value	*I* ^2^
VAS	–0.60 [–1.04, –0.16]	0.007	82.9% (*p* < 0.001)
AUSCAN	–0.31 [–1.73, 1.12]	0.674	94.6% (*p* < 0.001)
Grip strength	0.43 [0.10, 0.76]	0.010	60.9% (*p* = 0.026)
Pinch strength	0.52 [0.20, 0.84]	0.001	0% (*p* = 0.381)
HAQ	–0.57 [–0.82, –0.32]	0.000	3.4% (*p* = 0.387)
AUSCAN-pain	–0.46[–0.71, –0.21]	0.000	0% (*p* = 0.42)
AUSCAN-stiffness	–0.28 [–0.63, 0.07]	0.112	30.4% (*p* = 0.231)
AUSCAN-function	–0.23 [–0.59, 0.13]	0.204	32.1% (*p* = 0.225)

**Fig. 5:**
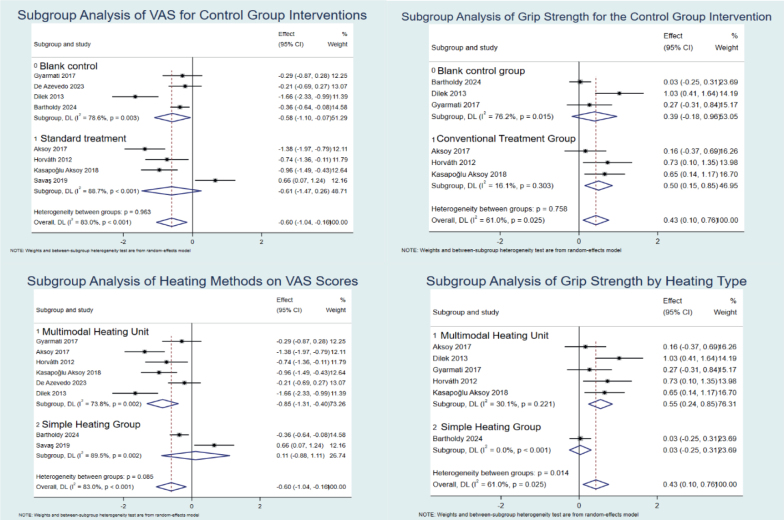
Forest plot for subgroup analysis.

Subgroup analysis revealed the potential impact of different heating methods and different control group interventions on treatment efficacy (Fig. 5). Subgroup analysis of heating methods indicated that the multimodal heating unit demonstrated a significant analgesic effect (SMD = −0.85; 95% CI: −1.31, −0.40). In contrast, the effect size for the monotherapy group was 0.11 (95% CI: −0.88, 1.11), which did not reach statistical significance. At the same time, multimodal heating significantly enhanced grip strength (SMD = 0.55; 95% CI: 0.24, 0.85). In contrast, the monotherapy group showed no significant improvement in grip strength (SMD = 0.03; 95% CI: –0.25, 0.31), and the difference between the 2 groups was statistically significant (*p* = 0.01). This indicates that the multimodal approach is superior to the monotherapy approach in terms of grip strength recovery. Subgroup analyses of the control group interventions indicated that, regardless of whether the control group consisted of a “no-intervention control” or “standard/conventional treatment”, heat therapy as a whole showed a trend towards reducing pain and improving grip strength. The intergroup comparison for VAS yielded a *p*-value of 0.963, and for grip strength a *p*-value of 0.758, suggesting that the design of the control group did not significantly alter the core benefits of heat therapy. To further investigate the potential sources of heterogeneity, we conducted a meta-regression analysis. The results are shown in Table III. The analysis revealed that only VAS scores and grip strength had sufficient studies to support the findings of the meta-regression analysis. The duration of a single treatment session (*p* = 0.03) and the number of weekly treatment sessions (*p* = 0.04) had a statistically significant effect on the heterogeneity of grip strength outcomes.

**Table III:** Results of meta-regression

Outcome indicators	Age	Sample size	T/Temperature	Duration of a single session	Number of sessions per week
VAS	0.30	0.13	0.98	0.92	0.17
Grip strength	0.14	0.127	0.446	0.03	0.04

At the same time, sensitivity analyses showed that the effect sizes for VAS, grip strength and HAQ remained largely consistent after excluding individual studies, indicating the robustness of the results. However, the effect sizes for the AUSCAN total score and grip strength showed some variation, suggesting that these results were less stable (Tables SII–SVI).

### Reporting bias assessment and certainty assessment

The Egger test was used to assess the risk of publication bias. The results showed that there was no publication bias for either VAS scores (*p* = 0.45) or grip strength (*p* = 0.055). Regarding grip strength, although *p* = 0.055 did not reach the conventional level of significance, given the low power of the analysis due to only 6 studies, this result suggests a possible small-sample effect (such as publication bias). The certainty of evidence for the primary outcome measures was assessed using the GRADE approach. The results showed that the certainty of evidence for VAS pain (SMD −0.60) and grip strength (SMD 0.43) was moderate (downgraded primarily due to risk of bias). The certainty of evidence for negative outcomes such as the AUSCAN total score and pinch strength was low (downgraded due to inconsistency and risk of bias). (Table SVII).

### Supplementary analysis

When *r* in the formula was set to 0.3 and 0.7 respectively, the conversion of the formula had no apparent effect on the results.

### Studies not included in the meta-analysis

In addition, there were 4 further studies, all of which were active-controlled trials. Due to excessive methodological heterogeneity, these were not included in the meta-analysis and are summarized here. Aksanyar (19), Öncel (28), and Uçar (30) examines the differences in efficacy between various heat therapies for patients with osteoarthritis of the hands. Ustun (31) compared the efficacy of prolotherapy and paraffin wax. Four active-controlled trials compared the efficacy of different heat therapies, and the results showed that pain improved in patients in all groups. This indirectly suggests the potential benefits of heat therapy; however, due to the absence of non-heat control groups, it is not possible to directly assess its absolute efficacy.

## DISCUSSION

This systematic review and meta-analysis, through the quantitative synthesis of existing randomized controlled trials, confirmed the significant clinical benefits of heat therapy in the short-term management of hand osteoarthritis (HOA). In summary, the findings indicate that this intervention not only demonstrates statistical superiority in alleviating pain as measured by VAS scores and the AUSCAN pain dimension, but also has a clear effect on improving grip strength and HAQ quality of life scores; however, the beneficial effects of heat therapy on indicators such as joint stiffness, pinch strength, and the AUSCAN functional total score are not yet significant, and this efficacy is influenced by variations in intervention modality and treatment dosage.

Intermittent pain, morning stiffness, and limited joint mobility are typical symptoms of osteoarthritis of the hands (1). Therefore, the ability to alleviate these typical symptoms is a key indicator in evaluating treatment options. This study demonstrates that patients experienced significant improvements in pain scores following heat therapy, prompting further investigation into the underlying mechanisms. Heat therapy not only downregulates the levels of pro-inflammatory cytokines (such as TNF-α, IL-1, and IL-6), thereby reducing stimulation of pain-sensing nerves and alleviating pain, but also reduces pain sensitivity by decreasing the expression of nerve growth factor (NGF) (32). Meanwhile, research by Cataldo et al. suggests that the inhibitory effect of thermal sensation on pain is a non-spatially specific, non-proportional regulatory mechanism; the specific mechanism may be related to descending regulatory systems in the brainstem (such as conditional pain modulation) (33). Heat therapy administered to patients with hand osteoarthritis not only reduced their overall perceived pain levels (decrease in VAS scores) but also specifically alleviated pain associated with specific daily hand activities (such as grasping and twisting) (decrease in AUSCAN-pain scores). This suggests that heat therapy is an effective approach capable of improving both “resting pain” and “functional pain” simultaneously.

In cases of morning stiffness and joint stiffness, patients with osteoarthritis of the hands often develop bony enlargements (nodules) on the dorsal aspects of the distal and proximal interphalangeal joints; these nodules result from subchondral bone remodelling and the formation of osteophytes. These are hard, bony protrusions. At the same time, osteoarthritis of the hands can lead to the wearing away of joint cartilage, as well as fibrosis and contracture of the joint capsule (34). It is the combined effect of these pathological changes that limits patients’ functional activity. As thermotherapy cannot reverse the processes of cartilage wear or bone spur formation, it is difficult to fundamentally improve symptoms such as morning stiffness and restricted joint mobility; consequently, no significant improvement was observed in the AUSCAN score, its sub-scores (stiffness and function), or grip strength.

This study observed significant improvements in HAQ scores following heat therapy, but no statistically significant differences were found in the AUSCAN-function dimension. This discrepancy is not coincidental, but rather reflects the fundamental differences between the 2 assessment tools in terms of the dimensions they capture and their disease-specific sensitivity. AUSCAN is specifically designed for hand osteoarthritis (HOA), whereas HAQ assesses systemic function. AUSCAN focuses strictly on hand-specific biomechanical movements (such as buttoning a shirt or wringing out a towel). Studies have shown that, in individuals with hand osteoarthritis, the physical function dimension of AUSCAN is more sensitive to local biomechanical changes; however, its minimum clinically important difference (MCID) is typically set more stringently than that of generalized scales (35). Relevant literature suggests that the HAQ may exhibit a “ceiling effect” when assessing hand-specific functions, or that its inclusion of non-hand-related items (such as walking and hygiene) may dilute its ability to capture the response to hand therapy (36).

At the clinical translation level, subgroup analyses and meta-regression results have further clarified the dose–response relationship and the relative merits of different modalities. Multimodal thermotherapy (such as paraffin wax baths and mud baths), by combining mechanical compression during the cooling process, chemical osmosis of minerals, and more efficient deep-tissue heat conduction, offers significantly greater clinical benefits than simple home-based dry heat interventions. Regression analysis confirmed that session duration and weekly intervention frequency are key moderators of grip strength recovery; this finding emphasizes that standardized treatment dosing is a prerequisite for achieving functional outcomes.

Although some studies could not be included in the meta-analysis due to methodological heterogeneity, the results of the narrative synthesis indicate that the qualitative findings of these studies (VAS scores, grip strength) are highly consistent with the results of the meta-analysis, which to some extent enhances the robustness of the conclusions of this review.

At the same time, complications associated with heat therapy are relatively rare; Bartholdy et al. reported only 3 adverse events, of which only 1 – involving itching caused by the use of a fingerless glove – was related to the heat therapy (21). Fioravanti et al. reported that 5% of patients experienced treatment-related adverse effects, but these were all mild and did not interrupt the course of treatment (24).

The results of this meta-analysis indicate that heat therapy, as a non-invasive intervention for hand osteoarthritis, not only effectively alleviates patients’ pain but also significantly improves grip strength. Therefore, heat therapy can be recommended as a safe and effective adjunctive analgesic strategy within comprehensive management programmes for hand osteoarthritis. In current clinical practice, although various medications and rehabilitation programmes are available for the treatment of osteoarthritis of the hand, there remains a pressing demand among patients for non-pharmacological therapies that are safe, convenient, and free from drug-related side effects. The conclusions of this study support the use of heat therapy as an effective symptomatic treatment. This not only helps to reduce patients’ reliance on oral analgesics but also provides clinicians with a new option for developing individualized rehabilitation programmes.

Compared with earlier reviews such as that by Ye et al. (14). or Kim et al. (10), which focused specifically on paraffin therapy, this study incorporates the latest evidence up to the end of 2025. It not only updates effect size estimates but also provides the first scientific evaluation of modern wearable heating technologies, establishing the high safety and efficacy of thermotherapy as a non-invasive management option for HOA. Although the short follow-up period and limited sample size preclude definitive conclusions regarding long-term effects on the evolution of bony structures, this analysis nevertheless provides crucial evidence-based support for the stepwise management of OA. It is recommended that clinical practice prioritize multimodal interventions to maximize clinical benefits, whilst ensuring appropriate treatment frequency.

However, this study has the following limitations. First, the relatively small number of included studies may result in insufficient statistical power. Second, the follow-up periods in most of the original studies were short, making it impossible to assess the long-term efficacy and long-term safety of hyperthermia therapy. Third, as the measurement tools for outcome indicators were not entirely consistent across studies, there may be some measurement bias. Fourth, although our subgroup analysis provided insights into the efficacy of different heat modalities, the limited number of trials and small cumulative sample size in certain subgroups may have restricted the statistical power to detect definitive differences. Thus, the observed superiority of multimodal therapy remains tentative. In summary, despite certain limitations, this meta-analysis provides positive evidence supporting the use of heat therapy in patients with hand osteoarthritis. As a safe, simple and non-invasive treatment option, heat therapy warrants further promotion and application in clinical practice.

In conclusion, moderate-to-low-quality evidence suggests that heat therapy (particularly multimodal approaches) is effective in alleviating short-term pain and improving grip strength in patients with osteoarthritis of the hands. Clinical practice should ensure adequate treatment frequency and duration; further high-quality studies with long-term follow-up are needed to verify its long-term efficacy.

## Supplementary Material






