# Supplementary material for: THE SHORT-TERM EFFICACY OF HEAT THERAPY FOR OSTEOARTHRITIS OF THE HANDS: A SYSTEMATIC REVIEW AND META-ANALYSIS
Source: J Rehabil Med. 2026 Aug 10;58:45883. doi: 10.2340/jrm.v58.45883 (PMC13463912; doi:10.2340/jrm.v58.45883)
Supplement: Supplementary file 1 [file JRM-58-45883-s1.pdf]

Supplementary material has been published as submitted. It has not been copyedited, or typeset by Journal of Rehabilitation Medicine

Appendix S1

$$M_{post} = M_{pre} + M_{diff}$$

$$SD_{post} = \sqrt{SD_{pre}^2 + SD_{diff}^2 + (-2r) \times SD_{pre} \times SD_{diff}}$$

M_{post} : Difference in mean scores at the end point ; M_{pre} : Difference in mean scores at baseline ;

M_{diff} : Difference in the mean of the changes ; SD_{post} : Standard deviation at the finish line ;

SD_{pre} : Standard deviation of the baseline ; SD_{diff} : Standard deviation of the change

r : Coefficient of correlation between the baseline value and the change

Appendix S2

$$X \approx \frac{q_1 + m + q_3}{3}$$

$$S \approx \frac{q_3 - q_1}{\eta(n)}$$

q_1 : first quartile ; q_3 : interquartile range ; m : median

X : mean ; S : standard deviation ; η: Correlation coefficient ; n : Sample size

Appendix S3

$$M_T = \frac{\sum_{i=1}^{k}(n_i \times M_i)}{N}$$

$$SD_T = \sqrt{\frac{\sum_{i=1}^{k}[(n_i - 1) \times SD_i^2 + n_i \times (M_i - M_T)^2]}{N - 1}}$$

k: Number of subgroups ; M_i : The mean for each subgroup ; SD_i: Standard deviation for each subgroup ;

n_i : Sample size for each subgroup ; N : Total sample size
