# Supplementary material for: THE SHORT-TERM EFFICACY OF HEAT THERAPY FOR OSTEOARTHRITIS OF THE HANDS: A SYSTEMATIC REVIEW AND META-ANALYSIS
Source: J Rehabil Med. 2026 Aug 10;58:45883. doi: 10.2340/jrm.v58.45883 (PMC13463912; doi:10.2340/jrm.v58.45883)
Supplement: Supplementary file 2 [file JRM-58-45883-s2.pdf]

Supplementary material has been published as submitted. It has not been copyedited, or typeset by Journal of Rehabilitation Medicine

Table SI. The specific search strategy

database	Search number	Query	Results
Cochrane Library	#1	(hand osteoarthritis OR "hand joints osteoarthritis" OR "finger osteoarthritis" OR "thumb osteoarthritis" OR "carpometacarpal osteoarthritis" OR "trapeziometacarpal osteoarthritis" OR ((osteoarthritis) AND ("hand" OR "finger" OR "thumb" OR "MCP" OR "PIP" OR "DIP" OR "CMC" OR"TMC"))	13017
	#2	(paraffin OR (paraffin wax) OR (paraffin bath*) OR (wax bath*) OR (warm paraffin) OR (mud bath*) OR (mud pack*) OR (peloid*) OR (fango) OR (fangotherap*) OR (thermotherap*) OR (thermal therap*) OR (heat therap*): OR (superficial heat) OR (hot pack*) OR (hot compress*): OR ((heat)*OR (thermal) OR (heating) OR (warmed) OR (warming)): OR (Thermotherapy) OR （Paraffin Bath）OR(Mud) OR [Balneotherapy])	7333
	#3	(randomized controlled trial) OR (randomized) OR (controlled clinical trial) OR (placebo) OR (trial) OR (random)	1750373
	#4	#1 AND #2 AND #3	229
PubMed	#1	(Hand osteoarthritis [Title/Abstract]) OR (Osteoarthritis of the hand [Title/Abstract]) OR (Osteoarthritis of the Hand [Title/Abstract]) OR (HOA[Title/Abstract])	3448
	#2	(Mud bath [Title/Abstract]) OR (Mud [Title/Abstract]) OR (hévíz mud [Title/Abstract]) OR (Paraffin bath [Title/Abstract]) OR (Paraffin wax bath[Title/Abstract]) OR (heated mittens [Title/Abstract])OR (Heat therapy [Title/Abstract])OR (Heating therapy [Title/Abstract]) OR (Heat treatment [Title/Abstract]) OR (Thermal therapy[Title/Abstract]) OR (Hot spring[Title/Abstract])	33347
	#4	#1 AND #2 AND #3	158
web of science	#1	(osteoarthritis) OR (degenerative joint disease) OR (arthrosis)	115114
	#2	(hand)OR (finger) OR(thumb) OR(interphalangeal)OR (DIP) OR (PIP) OR (MCP) OR (metacarpophalangeal) OR (carpometacarpal) OR (CMC) OR (trapeziometacarpal) OR (TMC) OR (first carpometacarpal) OR （base of thumb）	1187522
	#3	(paraffin)OR (paraffin wax)OR(paraffin bath)OR (wax bath)OR (wax therapy)OR (warm paraffin)OR (mud bath) OR (mud pack) OR(mud therapy) OR (peloid) OR (fango) OR (fango therapy)OR(balneotherapy)OR(thermotherapy)OR （thermal therapy)OR (heat therapy) OR(superficial heat)OR (hot pack) OR (hot compress) OR ((heat)OR (thermal) OR (heating) OR (warmed) OR(warming))	60516
	#4	(randomized controlled trial) OR (randomized) OR (controlled clinical trial) OR (placebo) OR (trial) OR (random)OR (single-blind) OR (double-blind) OR (crossover) OR (cross-over) OR (parallel group) OR (parallel trial)	1156653
	#5	#1 AND #2 AND #3 AND #4	55

Embase	#1	(hand osteoarthritis OR "hand joints osteoarthritis" OR "finger osteoarthritis" OR "thumb osteoarthritis" OR "carpometacarpal osteoarthritis" OR "trapeziometacarpal osteoarthritis" OR ((osteoarthritis) AND ("hand" OR "finger" OR "thumb" OR "MCP" OR "PIP" OR "DIP" OR "CMC" OR"TMC")))	24101
	#2	(paraffin)OR (paraffin wax)OR(paraffin bath)OR (wax bath)OR (wax therapy)OR (warm paraffin)OR (mud bath) OR (mud pack) OR(mud therapy) OR (peloid) OR (fango) OR (fangotherapy)OR(balneotherapy)OR(thermotherap)OR （thermal therapy)OR (heat therapy) OR(superficial heat)OR (hot pack) OR (hot compress) OR ((heat)OR (thermal) OR (heating) OR (warmed) OR(warming))	163647
	#3	(randomized controlled trial) OR (randomized) OR (controlled clinical trial) OR (placebo) OR (trial) OR (random)OR (single-blind) OR (double-blind) OR (crossover) OR (cross-over) OR (parallel group) OR (parallel trial)	4203039
	#4	#1 AND #2 AND #3	126

Table SII. Results of leave-one-out sensitivity analysis for VAS

Study removed	Total SMD (95% CI)
Aksoy et al.2017	-0.49 [-0.93, -0.50]
Bartholdy et al.2024	-0.64[-1.20, -0.09]
De Azevedo al. 2023	-0.66 [-1.17, -0.16]
Dilek et al.2013	-0.46[-0.88, -0.05]
Gyarmati et al.2017	-0.65[-1.14, -0.15]
Horváth et al.2012	-0.59 [-1.08, -0.09]
Kasapoğlu Aksoy et al.2018	-0.55[-1.04, -0.06]
Savaş et al.2029	-0.76 [-1.15, -0.38]

Table SIII. Results of leave-one-out sensitivity analysis for AUSCAN

Study removed	Total SMD (95% CI)
Aksoy et al.2017	-0.04 [-2.23, 2.33]
Kasapoğlu Aksoy et al.2018	0.10[-2.07, 2.28]
Savaş et al.2029	-1.06 [-1.45, -0.67]

Table SIV. Results of leave-one-out sensitivity analysis for Grip strength

Study removed	Study removed
Aksoy et al.2017	0.49[0.10,0.89]
Bartholdy et al.2024	0.55 [0.24,0.85]
Dilek et al.2013	0.31[0.02,0.60]
Gyarmati et al.2017	0.47 [0.08,0.86]
Horváth et al.2012	0.38 [0.03,0.74]
Kasapoğlu Aksoy et al.2018	0.39 [0.02,0.76]

Table SV. Results of leave-one-out sensitivity analysis for Pinch strength

Study removed	Total SMD (95% CI)
Aksoy et al.2017	0.68 [0.28, 1.08]
Horváth et al.2012	0.48 [-0.02, 0.98]
Kasapoğlu Aksoy et al.2018	0.39 [-0.02, 0.80]

Table SVI. Results of leave-one-out sensitivity analysis for HAQ

Study removed	Total SMD (95% CI)
Aksoy et al.2017	-0.50 [-0.77, -0.22]
Fioravanti 2014	-0.58 [-0.91, -0.25]
Gyarmati 2017	-0.63 [-0.91, -0.35]
Horváth et al.2012	-0.64 [-0.91, -0.37]
Kasapoğlu Aksoy et al.2018	-0.50 [-0.78, -0.22]

Table VII. The GRADE Summary of Findings for the Outcomes

Certainty assessment							№ of patients		Effect		Certainty	Importance
№ of studies	Study design	Risk of bias	Inconsistency	Indirectness	Imprecision	Other considerations	Heat Therapy	control	Relative (95% CI)	Absolute (95% CI)		
**VAS**												
9	randomised trials	serious[a]	serious[b]	not serious	not serious	none	321	304	-	SMD **0.60 lower** (1.04 lower to 0.16 lower)	⊕⊕◯◯ Low[a,b]	CRITICAL
**AUSCAN**												
4	randomised trials	serious[a]	serious[b]	not serious	not serious	none	119	104	-	SMD **0.31 lower** (1.73 lower to 1.12 higher)	⊕⊕◯◯ Low[a,b]	CRITICAL
**Grip strength**												
7	randomised trials	serious[a]	not serious	not serious	not serious	none	258	251	-	SMD **0. 43 higher** (0.10higher to 0.76 higher)	⊕⊕⊕◯ Moderate[a]	IMPORTANT
**Pinch strength**												
3	randomised trials	serious[a]	not serious	not serious	not serious	none	82	76	-	SMD **0.52 higher** (0.20 higher to 0.84 higher)	⊕⊕⊕◯ Moderate[a]	IMPORTANT
**HAQ**												
5	randomised trials	serious[a]	not serious	not serious	not serious	none	135	130	-	SMD **0.57 lower** (0.82 lower to 0.32 lower)	⊕⊕⊕◯ Moderate[a]	NOT IMPORTANT

CI: confidence interval; **SMD:** standardised mean difference

Explanations

a. The study has high risk

b. Some studies showed no significant differences between groups.
