# Supplementary material for: THE SHORT-TERM EFFICACY OF HEAT THERAPY FOR OSTEOARTHRITIS OF THE HANDS: A SYSTEMATIC REVIEW AND META-ANALYSIS
Source: J Rehabil Med. 2026 Aug 10;58:45883. doi: 10.2340/jrm.v58.45883 (PMC13463912; doi:10.2340/jrm.v58.45883)

Supplementary material has been published as submitted. It has not been copyedited, or typeset by Journal of Rehabilitation Medicine

Fig. S1. Risk of bias summary

Study ID	D1	D2	D3	D4	D5	Overall
Aksanyar 2022	+	+	+	+	+	+
Aksoy 2017	+	+	+	+	+	+
Bartholdy 2024	+	+	+	+	+	+
De Azevedo 2023	!	-	+	+	+	-
D lek 2013	+	+	+	+	+	+
Fioravanti 2014	+	+	+	+	+	+
Gyarmati 2017	+	!	+	+	+	!
Horv th 2012	+	+	+	+	+	+
Kasapoğlu Aksoy 2018	!	!	-	+	+	-
[illegible]ecel 2021	+	-	+	+	+	-
Savaş 2019	+	!	+	+	+	!
U[illegible]r 2011	!	-	+	+	+	-
Ustun 2023	+	!	+	+	+	!

D1 Rando2ization process

D2 Deviations from the intended interventions

D3 Missing outcome data

D4 Measurement of the outcome

D5 Selection of the reported result

+ Low risk

! Some concerns

- High risk

Fig. S2. Risk of bias graph

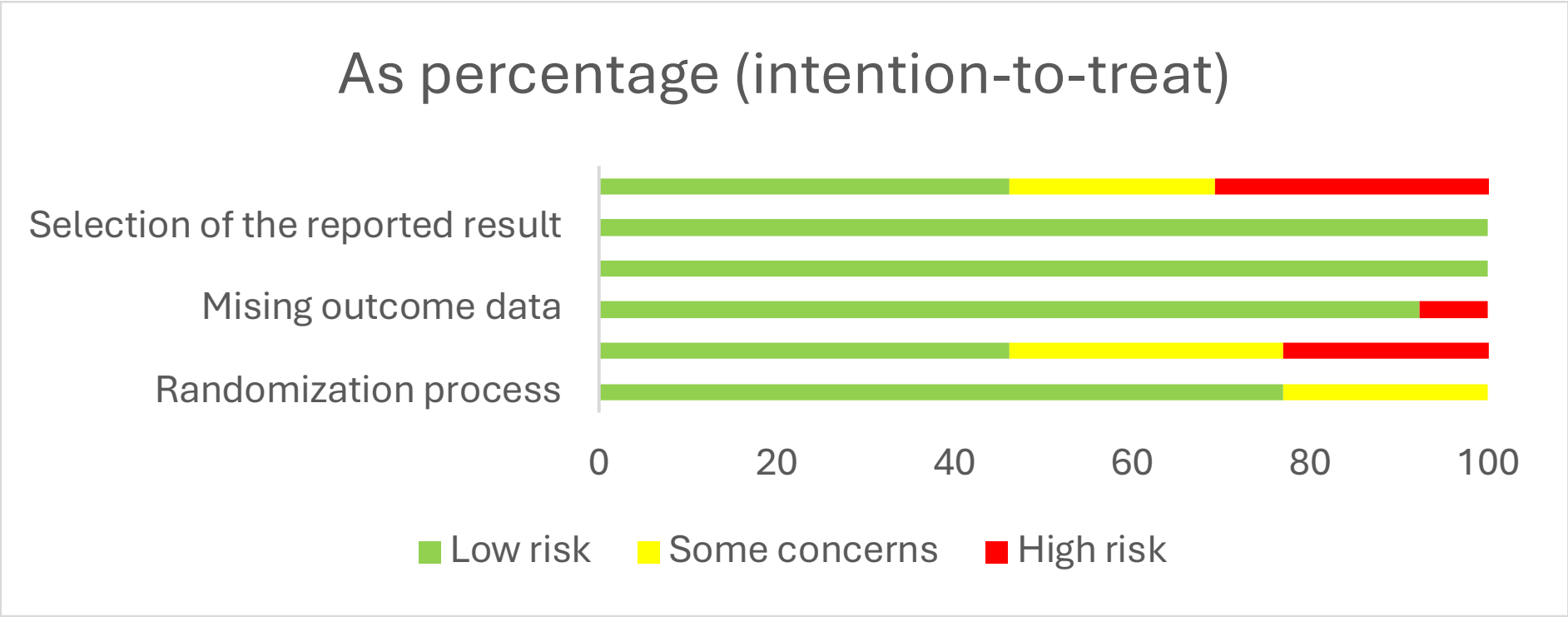

Supplement: Supplementary file 3 [file JRM-58-45883-s3.pdf]
